# Cardiogenic control of affective behavioural state

**DOI:** 10.1038/s41586-023-05748-8

**Published:** 2023-03-01

**Authors:** Brian Hsueh, Ritchie Chen, YoungJu Jo, Daniel Tang, Misha Raffiee, Yoon Seok Kim, Masatoshi Inoue, Sawyer Randles, Charu Ramakrishnan, Sneha Patel, Doo Kyung Kim, Tony X. Liu, Soo Hyun Kim, Longzhi Tan, Leili Mortazavi, Arjay Cordero, Jenny Shi, Mingming Zhao, Theodore T. Ho, Ailey Crow, Ai-Chi Wang Yoo, Cephra Raja, Kathryn Evans, Daniel Bernstein, Michael Zeineh, Maged Goubran, Karl Deisseroth

**Affiliations:** 1 Department of Bioengineering, Stanford University, Stanford, CA USA; 2 Department of Pediatrics, Stanford University, Stanford, CA USA; 3 Department of Radiology, Stanford University, Stanford, CA USA; 4 Department of Psychiatry and Behavioral Sciences, Stanford University, Stanford, CA USA; 5 Howard Hughes Medical Institute, Stanford University, Stanford, CA USA

**Keywords:** Insula, Cardiovascular biology

## Abstract

Emotional states influence bodily physiology, as exemplified in the top-down process by which anxiety causes faster beating of the heart^1–3^. However, whether an increased heart rate might itself induce anxiety or fear responses is unclear^3–8^. Physiological theories of emotion, proposed over a century ago, have considered that in general, there could be an important and even dominant flow of information from the body to the brain^9^. Here, to formally test this idea, we developed a noninvasive optogenetic pacemaker for precise, cell-type-specific control of cardiac rhythms of up to 900 beats per minute in freely moving mice, enabled by a wearable micro-LED harness and the systemic viral delivery of a potent pump-like channelrhodopsin. We found that optically evoked tachycardia potently enhanced anxiety-like behaviour, but crucially only in risky contexts, indicating that both central (brain) and peripheral (body) processes may be involved in the development of emotional states. To identify potential mechanisms, we used whole-brain activity screening and electrophysiology to find brain regions that were activated by imposed cardiac rhythms. We identified the posterior insular cortex as a potential mediator of bottom-up cardiac interoceptive processing, and found that optogenetic inhibition of this brain region attenuated the anxiety-like behaviour that was induced by optical cardiac pacing. Together, these findings reveal that cells of both the body and the brain must be considered together to understand the origins of emotional or affective states. More broadly, our results define a generalizable approach for noninvasive, temporally precise functional investigations of joint organism-wide interactions among targeted cells during behaviour.

## Abstract (web summary)

Direct elevation of heart rate using noninvasive optogenetics in mice influences anxiety-like behaviours in specific environmental contexts, and the posterior insular cortex is implicated in this integration of signals from the heart with environmental risk information.

## Main

Interoceptive processing of visceral physiological signals, such as cardiac palpitations or stomach fullness, is crucial for maintaining homeostasis^1–3^. Diverse psychiatric conditions, such as anxiety disorders, panic disorder, body dysmorphic disorders and addiction, have been hypothesized to be related to dysregulation of interoceptive monitoring by the brain^3,4^, and can be statistically correlated with specific visceral organ dysfunction. For example, patients with panic disorder and agoraphobia are more likely to have mitral valve prolapse or clinical symptoms similar to paroxysmal supraventricular tachycardia^5,6^. Modern correlative studies have further suggested links between cardiac changes and affect regulation^7,8^, including correlations between cardiac interoception with anxiety and functional alterations in the insular cortex, a cortical region that has a central role in both the processing of physiological signals and the regulation of emotions^4,10^. However, determining whether primary physiological signals such as increased heart rate can causally influence behavioural states, as proposed in classical physiological theories of emotion^9^, has—although widely debated—remained largely experimentally intractable^11^. Available nonspecific interventions that might disrupt cardiac signals (such as electrical vagus nerve stimulation) are well known to also induce numerous physiological changes that would be unwanted in this context, including direct suppression of respiratory and heart rates as well as anxiolytic and antidepressive effects^11–13^, giving rise to multiple direct confounds for the question explored here. Other nonspecific interventions to alter cardiac rhythms, such as broadly active pharmacological stimulants or electrical pacemakers^14^, also introduce insuperable confounds through initial actions beyond the direct pacing of cardiomyocytes, and thus lack the necessary precision. Studying the key question of how cardiac physiology regulates emotional states has remained inaccessible, and the effects on behaviour remain unknown.

Precise modulation of electrochemical signals in the heart and other peripheral organs in vivo would enable fundamental studies of physiology and interoceptive signalling^15–19^, but stimulation methods that operate with high spatial and temporal precision in highly dynamic environments such as the beating heart^20–28^ are limited. Electrical pacemakers require invasive surgical implantation to deliver local indiscriminate stimulation that lacks cell-type specificity^20–23^. Optogenetics might in principle facilitate cardiomyocyte-specific control with high spatial and temporal precision^14^, but existing optogenetic methods have been limited to acute demonstrations that require exposure or even excision of the heart to deliver light^23–28^, all of which are incompatible with freely moving studies of behaviour. Thus far, to our knowledge, no study beyond the brain has achieved precise and noninvasive organ-level control of behavioural or physiological function. Establishing noninvasive approaches to manipulate physiology with cell-type specificity would enable long-sought functional studies of signals that arise from cells across the organism, and reveal the causal influences of these cells on brain function and behaviour.

Conventional microbial opsins have not been sensitive enough to control a large organ such as the heart with the requisite power to facilitate behavioural studies within intact animals^23–28^, but the discovery of the highly sensitive and red-shifted pump-like channelrhodopsin ChRmine led us to consider the potential for noninvasive optogenetic control of deep tissue with minimal irradiance^29^. We previously found that ChRmine enabled neuromodulation of deep brain circuits without intracranial surgery^30^, which raised the possibility that this optogenetic tool might be broadly applicable to modulating biological processes across the entire body of large organisms such as mammals. Specifically, we hypothesized that ChRmine might allow on-demand deep-tissue control of cardiac pacing when targeted to cardiomyocytes without the need for direct exposure of the heart^29,30^.

## Noninvasive optogenetic cardiac control

**Extended Data Fig. 1:**
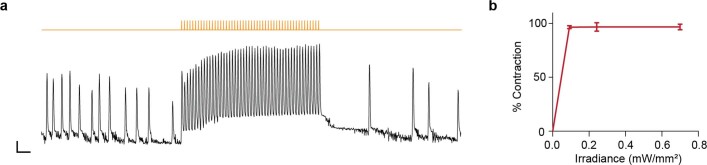
In vitro characterization of the optical pacemaker. **a**, Quantified cardiomyocyte contraction sequence measured by centroid motion (see Supplementary Video 1). Optical stimulation was applied at 5 Hz, with 10-ms pulse width at 585 nm. Scale: 1 s, 1 pixel. **b**, Fidelity of optically induced contractions at different light intensities. n = 20 cells. Data represent mean ± s.e.m.

We first achieved cardiomyocyte-restricted expression by placing the ChRmine transgene under the control of the mouse cardiac troponin T promoter (mTNT), using the AAV9 serotype, which exhibits tropism for cardiac tissue^25,31^. Infection of cultured primary cardiomyocytes with AAV9-mTNT::ChRmine-2A-oScarlet enabled light-evoked contractions with irradiance as low as 0.1 mW mm^−2^, consistent with the photosensitivity of ChRmine in neurons^29^ (Extended Data Fig. 1 and Supplementary Video 1).

**Fig. 1:**
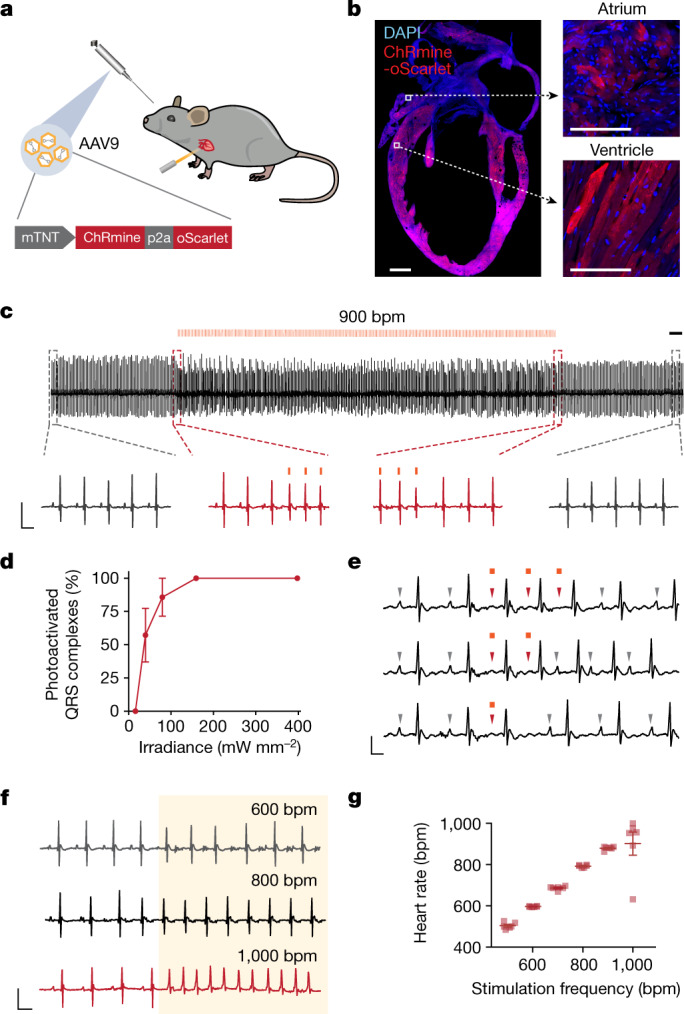
Development of a noninvasive optical pacemaker. **a**, Schematic showing the optical control of cardiac rhythm with an external light source enabled by retro-orbital injection of AAV9-mTNT::ChRmine-p2A-oScarlet. **b**, Confocal cross-section images indicating homogeneous transgene expression of ChRmine-p2A-oScarlet (red) with DAPI staining (blue) in atria and ventricles. Scale bars, 1 mm (main); 100 µm (inset). **c**, Example electrocardiogram (ECG) trace with optical pacing using 589 nm light delivered at 15 Hz (at 900 bpm) with a pulse width of 10 ms and irradiance of 160 mW mm^−2^. Scale bar, 500 ms. Inset traces: ECG signal before and after light delivery (grey) and at light onset and cessation (red). Scale bar, 50 ms, 0.5 mV. **d**, Reliability of photoactivated QRS complexes at 900 bpm as a function of cutaneous optical irradiance (*n* = 6 mice). **e**, Example ECG traces of individual 10-ms optical pulses. Grey arrowheads indicate P waves associated with sinus rhythm, which are overridden (red arrowheads) during optical pacing. Scale bar, 25 ms, 0.25 mV. **f**, Example ECG traces of pacing at 600, 800 and 1,000 bpm. Scale bar, 50 ms, 0.5 mV. The shaded area indicates the period of illumination at the specified frequency at 100% duty cycle. **g**, Characterization of optical pacing fidelity, showing stimulation frequency versus ECG-measured heart rate (*n* = 6 mice). All ECG measurements were performed in anaesthetized mice. Data are mean ± s.e.m.

**Extended Data Fig. 2:**
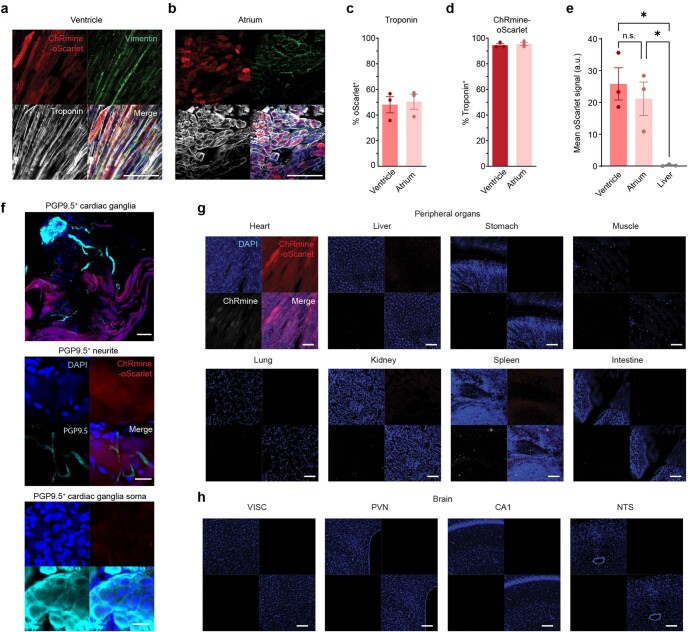
In vivo characterization of AAV9-mTNT::ChRmine-2A-oScarlet. **a**,**b**, Representative confocal images of ChRmine-2A-oScarlet (red)-infected ventricular (**a**) and atrial (**b**) cardiac tissue, co-labelled with troponin (white), vimentin (green), and DAPI (blue). Note ChRmine expression is restricted to troponin^+^ cardiomyocytes with no off-target labelling in neighbouring vimentin^+^ fibroblasts. Scale bar=100 µm. **c**, Penetrance of AAV9-mTNT::ChRmine-p2A-oScarlet quantified as percentage of troponin^+^ cells that express ChRmine-2A-oScarlet (n = 3). **d**, Specificity of AAV9-mTNT::ChRmine-p2A-oScarlet quantified as percentage of ChRmine-2A-oScarlet^+^ cells that are troponin^+^ (n = 3). **e**, Quantification of oScarlet expression in ventricular and atrial cardiac tissue and liver as mean fluorescence signal (arbitrary unit) following 1 month post-injection of AAV9-mTNT::ChRmine-p2A-oScarlet (n = 3, one-way repeated-measures ANOVA with Bonferroni post-hoc test: F(2,4)=18.3, p = 0.0097. Post-hoc: ventricle vs. atrium p = 0.99, ventricle vs. liver p = 0.014, atrium vs liver p = 0.029). **f**, Representative confocal image from one of two mice depicting lack of neuronal labelling in cardiac ganglia following retro-orbital delivery of  AAV9-mTNT::ChRmine-p2A-oScarlet as measured by immunostaining for the neural marker PGP9.5 (cyan). No off-target ChRmine expression was observed in neurites (centre) or in the soma of cardiac ganglia (bottom). Scale bar=100 µm (top), 20 µm (center, bottom). **g**,**h**, Representative confocal images of oScarlet (red), ChRmine mRNA (white) and nuclei (DAPI) nuclei across different organs (**g**) after 9 months of expression from one of two mice. No off-target expression of ChRmine was observed in organs beyond the heart, including throughout the brain (**h**). Scale bar=100 µm. Data represent mean ± s.e.m.

**Extended Data Fig. 3:**
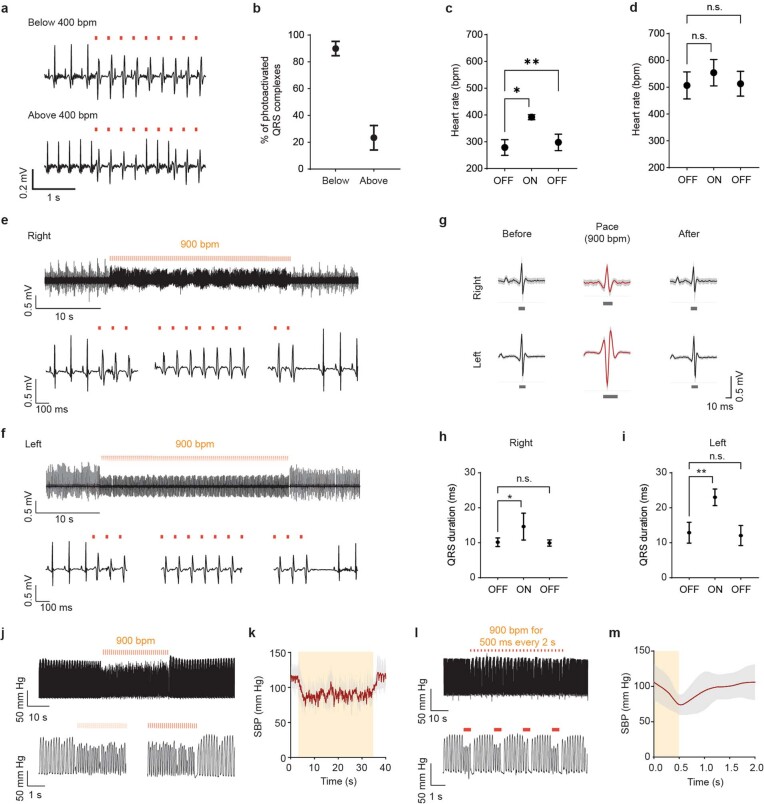
Quantification of cardiac responses to optical pacing in vivo. **a**, Representative ECG recording with optical pacing at 400 bpm below (top) or above (bottom) a resting heart rate of 400 bpm. **b**, Percentage of photoactivated heart beats that track with the delivered 400 bpm optical stimulus below or above a resting heart of 400 bpm (n = 4 (below), n = 5 (above) mice). **c**, Measured heart rate before, during, and after pacing at 400 bpm in heavily anesthetized mice with a resting heart rate below 400 bpm (n = 4 mice, one-way repeated-measures ANOVA with Bonferroni post-hoc test: condition F_(1.01, 3.03)_=34.07, p = 0.0097; individual F_(3,6)_=2.4, p = 0.17. Post-hoc: OFF vs. ON *p = 0.015, OFF vs. OFF **p = 0.002). **d**, Measured heart rate before, during, and after pacing at 400 bpm in lightly anesthetized mice with a resting heart rate above 400 bpm (n = 5 mice, one-way repeated-measures ANOVA with Bonferroni post-hoc test: condition F_(1.06, 4.24)_=3.71, p = 0.12; individual F_(4,8)_=5.9, p = 0.016. Post-hoc: OFF vs. ON p = 0.17, OFF vs. OFF p = 0.72). **e**, Representative ECG recording with optical pacing delivered at 900 bpm with 10-ms pulse width with laser positioned at the centre of the chest to induce right ventricular pacing (top) with additional traces from before, during, and after light stimulation (bottom). **f**, Representative ECG recording with optical pacing delivered at 900 bpm with 10-ms pulse width with laser positioned more lateral of the chest to induce left ventricular pacing (top) with additional traces from before, during, and after light stimulation (bottom). **g**, Representative QRS complexes averaged over 100 heart beats before, during, and after pacing for right and left ventricular stimulation. Dark grey bar indicates duration of QRS complex. **h**, QRS duration before, during, and after right ventricular pacing (n = 4 mice, one-way repeated-measures ANOVA with Bonferroni post-hoc test: condition F_(1.01, 3.04)_=25.4, p = 0.015; individual F_(3,6)_=0.68, p = 0.59. Post-hoc: OFF vs. ON *p = 0.037, OFF vs. OFF p = 0.99). **i**, QRS duration before, during, and after left ventricular pacing (n = 4 mice, one-way repeated-measures ANOVA with Bonferroni post-hoc test: condition F_(1.18, 3.54)_=121.7, p = 6.7e-4; individual F_(3,6)_=16.64, p = 0.0026. Post-hoc: OFF vs. ON **p = 0.0038, OFF vs. OFF p = 0.20). **j**, Representative left ventricular blood pressure recordings with sustained optical pacing delivered at 900 bpm with 10-ms pulse width (top) with additional traces from before and after light stimulation (bottom). **k**, Systolic blood pressure (SBP) over time with sustained 900 bpm optical pacing (orange) for 30 s showing a sustained drop in SBP during stimulation (n = 3 mice). **l**, Representative left ventricular blood pressure recordings with intermittent optical pacing delivered at 900 bpm with 10-ms pulse width for 500 ms every 1,500 ms (top) with additional traces during light stimulation (bottom). **m**, Averaged SBP over the interval of optical stimulation (500 ms ON at 900 bpm, 1,500 ms OFF) (n = 3 mice). Data represent mean ± s.d.

We next determined whether systemic viral gene delivery of ChRmine, despite the lower multiplicity of infection compared with transduction by direct local injection^30,32^, could allow noninvasive control of heart rhythms in wild-type mice. Retro-orbital injection of AAV9 enabled restricted expression of ChRmine in cardiomyocytes throughout the heart, with homogeneous expression in both ventricular and atrial walls and no off-target expression in other cardiac cell types (fibroblasts and neuronal ganglia) or in other organs (Fig. 1a,b and Extended Data Fig. 2). When pulsed 589-nm light was delivered through intact skin overlying the thorax of anaesthetized mice, we observed robust photoactivation of cardiac QRS complexes within a safe range of irradiance comparable to that used for transcranial optogenetics^30^ (Fig. 1c,d). Reliable cardiac rhythms were induced at even supraphysiological rates of up to 900 beats per minute (bpm), within the photophysical properties of ChRmine^33^, with an immediate return to naturally paced sinus rhythm upon the cessation of light (Fig. 1e–g). This approach was not able to decrease heart rate below baseline levels, but afforded spatial control of cardiac rhythms by evoking either right or left ventricular pacing depending on the placement of the laser (Extended Data Fig. 3a–i).

**Fig. 2:**
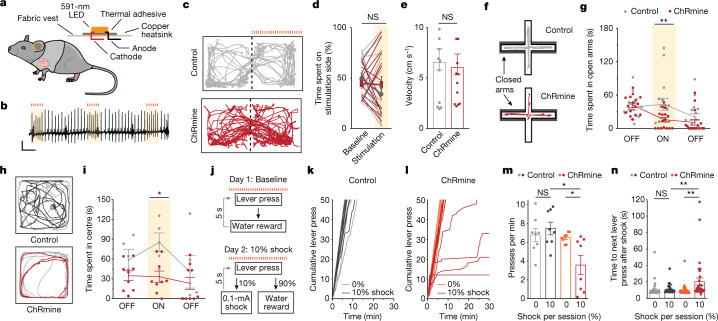
Optically induced tachycardia increases anxiety-like behaviour. **a**, Schematic of a micro-LED mounted to a wearable vest and fastened onto a mouse. **b**, Representative ECG trace of optically induced tachycardia (900 bpm for 500 ms every 2 s) used for all behavioural assays. Scale bar, 0.2 mV, 500 ms. **c**, Example path trace of a mouse with (red) or without (grey) ChRmine expression during an RTPP test, in which mice received optically induced cardiac pacing on one side of the chamber. **d**, Percentage of time spent on stimulation side during baseline and stimulation days for control (grey) and ChRmine-expressing (red) mice (*n* = 16 mice per group; two-way repeated-measures ANOVA with Bonferroni post hoc test: group (opsin) × time interaction *F*_(1,30)_ = 2.29, *P* = 0.14; group (opsin) effect *F*_(1,30)_ = 6.2 × 10^−4^, *P* = 0.98; time effect *F*_(1,30)_ = 2.06, *P* = 0.16. Bonferroni post hoc: control versus ChRmine *P* = 0.71 (baseline day); *P* = 0.67 (stimulation day)). NS, not significant. **e**, Average velocity on the optically paced side during RTPP (*n* = 16 mice per group; unpaired two-tailed *t*-test, *P* = 0.81). **f**, Example path trace of control (grey) and ChRmine-expressing (red) mice during an EPM test with optical pacing during the 5-min ON epoch of a 15-min trial. Open arms are vertical; closed arms are horizontal and bordered in grey. **g**, Time spent in open arms during 5-min epochs of EPM exploration (*n* = 16 mice per group; two-way repeated-measures ANOVA with Bonferroni post hoc test: group (opsin) × time interaction *F*_(2,60)_ = 3.906, *P* = 0.0254; group (opsin) effect *F*_(1,30)_ = 3.297, *P* = 0.0794; time effect *F*_(2,60)_ = 9.75, *P* = 0.0002. Bonferroni post hoc: ON epoch ChRmine versus control, **P = 0.0079). **h**, Example path trace of control (grey) and ChRmine-expressing (red) mice during an OFT with optical pacing during the 3-min ON epoch of a 9-min trial. **i**, Time spent in the centre during 3-min epochs of OFT exploration (*n* = 5 (control), 9 (ChRmine) mice; two-way repeated-measures ANOVA with Bonferroni post hoc test: group (opsin) × time interaction *F*_(2,24)_ = 1.531, *P* = 0.024; group (opsin) effect *F*_(1,12)_ = 5.69, *P* = 0.0035; time effect *F*_(2,24)_ = 3.42, *P* = 0.049. Bonferroni post hoc: ON epoch ChRmine versus control, *P = 0.018). **j**, Vogel conflict task to assay for cardiogenic effects on operant behaviour. Water-restricted mice were first trained for 2–3 weeks until each mouse was able to complete the 50 water-reward lever-press trials over 30 min for at least 3 consecutive days. On the day of the behavioural task, mice received optical pacing while completing a total of 50 lever-press trials per session (day 1). On the subsequent day, a 10% pseudorandom chance of shock was introduced upon lever press (day 2). **k**,**l**, Cumulative lever presses during 0% (day 1) and 10% shock (day 2) sessions for control (**k**) and ChRmine-expressing (**l**) mice (*n* = 8 mice). **m**, Average lever-pressing rate during 0% baseline or 10% shock trial sessions (*n* = 8 mice per group; two-way repeated-measures ANOVA with Bonferroni post hoc test: group (opsin) × condition (shock) interaction *F*_(1,14)_ = 8.326, *P* = 0.0120; group (opsin) effect *F*_(1,14)_ = 7.39, *P* = 0.0166; condition (shock) effect *F*_(1,14)_ = 3.162, *P* = 0.0971. Bonferroni post hoc: control 0% versus 10%, *P* = 0.8933; ChRmine 0% versus 10%, *P = 0.0106; 0% control versus ChRmine, *P* > 0.9999; 10% control versus ChRmine, **P = 0.0010). **n**, Elapsed time between a lever press resulting in shock and a subsequent lever press as a measure of the mouse’s apprehension state. On 10% shock trials, 3 out of 8 mice did not complete the trial, so the time to next lever press for some trials cannot be measured (*n* = 40, 40, 40 and 32 presses per group in control 0%, control 10%, ChRmine 0% and ChRmine 10%; two-way ANOVA with Bonferroni post hoc test: group (opsin) × condition (shock) interaction *F*_(1,148)_ = 6.478, *P* = 0.0119; group (opsin) effect *F*_(1,148)_ = 5.041, *P* = 0.0262; condition (shock) effect *F*_(1,148)_ = 7.253, *P* = 0.0079. Bonferroni post hoc: control 0% versus 10%, P > 0.9999; ChRmine 0% versus 10%, **P = 0.0026; 0% control versus ChRmine, *P* > 0.9999; 10% control versus ChRmine, **P = 0.0074). Data are mean ± s.e.m.

**Extended Data Fig. 4:**
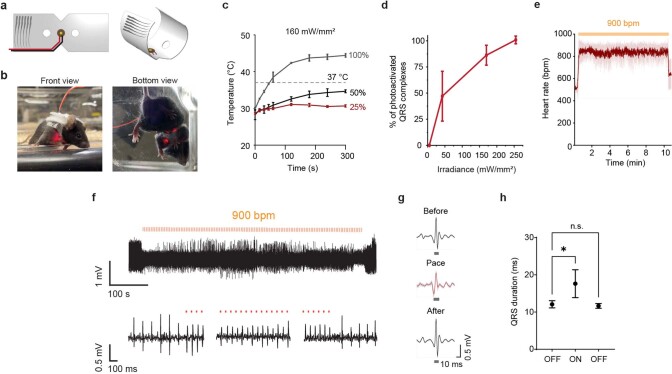
Characterization of the wearable micro-LED vest. **a**, Schematic of optical pacing vest. **b**, Photographs of freely moving mouse wearing optical pacing vest while receiving 591 nm light stimulation. **c**, Heating of device operated at 900 bpm with 10-ms pulse width at varying duty cycles (n = 3 devices, 25% (500 ms ON, 1,500 ms OFF), 50% (1 s ON, 1 s OFF), 100% (constant ON)). **d**, Percentage of photoactivated QRS complexes as a function of irradiance using the micro-LED device (n = 5). **e**, Measured heart rate across time over a 10 min stimulation period (900 bpm) showing ability of optical pacing to induce a stable heart rhythm (n = 6 mice). **f**, Representative ECG recording from one mouse receiving 900-bpm stimulation for 10 min (top) with additional traces from before, during, and after light stimulation (bottom). **g**, Representative QRS complexes averaged over 100 heart beats before, during and after pacing. **h**, QRS duration before, during and after right ventricular pacing (n = 5 mice, one-way repeated-measures ANOVA with Bonferroni post-hoc test: condition F_(1.03, 4.10)_=13.74, p = 0.02; individual F_(4,8)_=1.83, p = 0.21. Post-hoc: OFF vs. ON *p = 0.024, OFF vs. OFF p = 0.88). Data represent mean ± s.e.m.

To translate this approach to mouse behaviour, we mounted a 591-nm micro-LED onto a wearable fabric vest to deliver light through intact skin overlying the chest wall (Fig. 2a and Extended Data Fig. 4a,b). This integration of a molecular tool with accessible electronics enabled the initial demonstration of noninvasive and sustained ventricular pacing at experimenter-defined rhythms suitable for most behavioural assays in freely moving mice (Extended Data Fig. 4).

**Extended Data Fig. 5:**
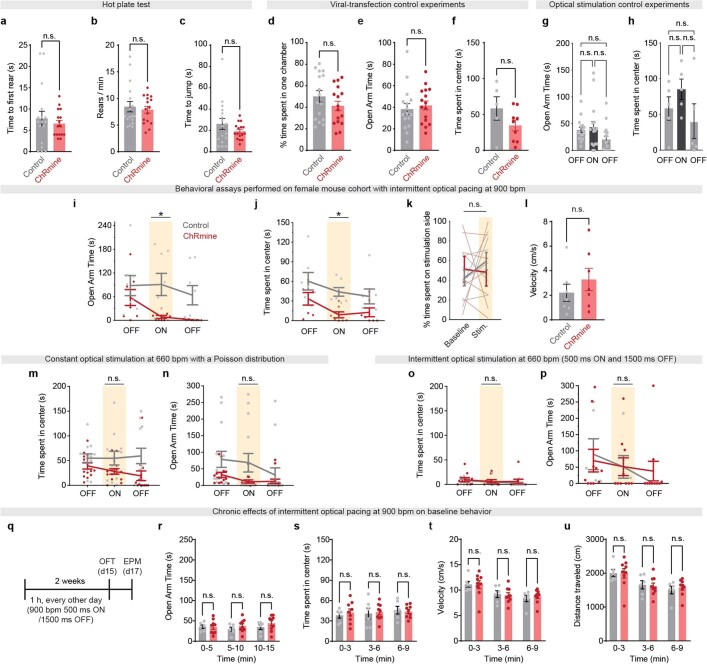
Additional characterization of optical-pacing effects on mouse behaviour. **a**–**c**, A hot-plate test was performed to assess for potential effects on thermal pain thresholds from optical pacing (n = 17 (control), 16 (ChRmine)) and the following were quantified: time to first rear (unpaired two-tailed t-test, p = 0.53) (**a**); rears per minute (unpaired two-tailed t-test, p = 0.64) (**b**); and time to first jump (unpaired two-tailed t-test, p = 0.22) (**c**). Note no statistical significance in thermal thresholds was observed with cardiac pacing. **d**–**f**, To assess behavioural differences between control and virally transduced ChRmine-expressing mice, the following comparisons were performed: time spent in one chamber during baseline day (no light delivery) (n = 16 per group, unpaired two-tailed t-test p = 0.21) (**d**); time spent in the open arm of the EPM test during the first 5-min epoch with no light delivery (n = 16 per group, unpaired two-tailed t-test p = 0.61) (**e**); and time spent in the centre of the OFT during the first 3-min epoch with no light delivery (n = 5 (control), 9 (ChRmine), unpaired two-tailed t-test p = 0.15) (**f**). Note no statistical significance in behaviour was observed from viral-transfection. **g**,**h**, To assess for effects of light stimulation alone on mouse behaviour, the following comparisons were performed: time spent in the open arms by control (saline injected) mice during the 15 min EPM assay, where intermittent light delivery (10-ms pulse width, 900 bpm for 500 ms every 2 s) was delivered during the 5 min ON epoch (n = 16, one-way repeated-measures ANOVA with Bonferroni post-hoc test: condition *F*_(1.44,21.62)_=2.942, p = 0.088, individual *F*_(15,30)_=1.61, p = 0.13. Post-hoc: OFF vs. ON p = 0.99, OFF vs. OFF p = 0.9, ON vs. OFF p = 0.27) (**g**); and time spent in the centre by control mice during the 9 min OFT, where intermittent light delivery was delivered during the 3 min ON epoch (n = 5, one-way repeated-measures ANOVA with Bonferroni post-hoc test: condition *F*_(1.84,7.45)_=1.67, p = 0.25, individual *F*_(4,8)_=1.51, p = 0.29. Post-hoc: OFF vs. ON p = 0.99, OFF vs. OFF p = 0.99, ON vs. OFF p = 0.41) (**h**). Note no statistical significance in behaviour was observed from light stimulation alone. **i**,**j**, To assess for effects from optical pacing (10-ms pulse width, 900 bpm for 500 ms every 2 s) on anxiety-like behaviour in female mice, the following behavioural assays were measured: time spent in the open arms during the EPM (n = 8 (control) and 7 (ChRmine) female mice, two-way repeated-measures ANOVA with Bonferroni post-hoc test: group (opsin) x time interaction *F*_(2,26)_=1.91, p = 0.17, group (opsin) effect *F*_(1,13)_=5.1, p = 0.042; time effect *F*_(2,26)_=4.24, p = 0.026. Bonferroni post-hoc: ON epoch ChRmine vs Control *p = 0.033) (**i**); and time spent in the centre during the OFT (n = 7 (control) and 7 (ChRmine) female mice, two-way repeated-measures ANOVA with Bonferroni post-hoc test: group (opsin) x time interaction *F*_(2,24)_=0.37, p = 0.70; group (opsin) effect *F*_(1,12)_=7.47, p = 0.018; time effect *F*_(2,24)_=6.9, p = 0.0043. Post-hoc: ON epoch ChRmine vs Control *p = 0.031) (**j**). **k**,**l**, To determine whether cardiac pacing was aversive in female mice, we also measured the percentage of time spent on baseline and stimulation day for control (grey) and ChRmine-expressing (red) mice during the RTPP assay (n = 7 female mice per group, two-way repeated-measures ANOVA with Bonferroni post-hoc test: group (opsin) x treatment interaction *F*_(1,12)_=0.68, p = 0.42; group (opsin) effect *F*_(1,12)_=0.11, p = 0.91; treatment effect *F*_(1,12)_=0.35, p = 0.57. Post-hoc: Baseline vs Stimulation for Control: p = 0.67, ChRmine: p = 0.99) (**k**); and the average velocity on the optically paced side during RTPP (n = 7 female mice per group, two-way repeated-measures ANOVA with Bonferroni post-hoc test: group (opsin) x treatment interaction *F*_(1,12)_=0.088, p = 0.77; group (opsin) effect *F*_(1,12)_=3.69, p = 0.079; treatment effect *F*_(1,12)_=0.12, p = 0.73. Post-hoc: Stimulation day Control vs Chrmine: p = 0.59) (**l**). **m**,**n**, To determine whether increased heart rate variability can affect anxiety-like behaviour, we introduced constant 660 bpm stimulation with a Poisson distribution in mice and measured the time spent in the centre during the OFT (n = 12 (control) and 14 (ChRmine) mice, two-way repeated-measures ANOVA with Bonferroni post-hoc test: group (opsin) x time interaction *F*_(2,48)_=1.29, p = 0.28; group (opsin) effect *F*_(1,24)_=5.80, p = 0.024; time effect *F*_(1.36,32.6)_=0.47, p = 0.55. Post-hoc ChRmine vs Control: OFF p = 0.61, ON p = 0.28, OFF p = 0.12) (**m**); and the time spent in the open arms during the EPM (n = 14 (control) and 14 (ChRmine) mice, two-way repeated-measures ANOVA with Bonferroni post-hoc test: group (opsin) x time interaction *F*_(2,52)_=1.75, p = 0.18; group (opsin) effect *F*_(1,26)_=3.3, p = 0.082; time effect *F*_(1.84,47.8)_=5.157, p = 0.011. Post-hoc ChRmine vs Control: OFF p = 0.25, ON p = 0.20, OFF p = 0.99) (**n**). **o**,**p**, To determine whether intermittent tachycardia at a rhythm below 900 bpm can affect anxiety-like behaviour, we introduced intermittent 660 bpm stimulation (10-ms pulse width, 660 bpm for 500 ms every 2 s) in mice and measured the time spent in the centre during the OFT (n = 6 (control) and 10 (ChRmine) mice, two-way repeated-measures ANOVA with Bonferroni post-hoc test: group (opsin) x time interaction *F*_(2,28)_=0.13, p = 0.88; group (opsin) effect *F*_(1,14)_=0.25, p = 0.63; time effect *F*_(2,28)_=1.1, p = 0.35. Post-hoc ChRmine vs Control: OFF p = 0.99, ON p = 0.99, OFF p = 0.99) (**o**); and the time spent in the open arms during the EPM (n = 6 (control) and 10 (ChRmine) mice, two-way repeated-measures ANOVA with Bonferroni post-hoc test: group (opsin) x time interaction *F*_(2,28)_=0.52, p = 0.60; group (opsin) effect *F*_(1,14)_=0.03, p = 0.86; time effect *F*_(2,28)_=2.23, p = 0.12. Post-hoc ChRmine vs Control: OFF p = 0.99, ON p = 0.99, OFF p = 0.99) (**p**). **q**, Schematic overview of the chronic stimulation experiment. Mice were stimulated with intermittent optical pacing (900 bpm for 500 ms every 2 s) for 1 h every other day for two weeks before performing the OFT and EPM behavioural assays. **r**, Time spent in open arm during the EPM test for control (grey) and ChRmine (red) mice subjected to chronic optical stimulation (n = 6 (control) and 9 (ChRmine) mice, two-way repeated-measures ANOVA with Bonferroni post-hoc test: group (opsin) x time interaction *F*_(2,26)_=0.87, p = 0.43; group (opsin) effect *F*_(1,13)_=0.71, p = 0.41; time effect *F*_(1.65,21.4)_=0.96, p = 0.38. Post-hoc ChRmine vs Control: 0–5 min p = 0.99, 5–10 min p = 0.89, 10–15 min p = 0.84). **s**, Time spent in centre during the OFT test for control (grey) and ChRmine (red) mice subjected to chronic optical stimulation (n = 6 (control) and 9 (ChRmine) mice, two-way repeated-measures ANOVA with Bonferroni post-hoc test: group (opsin) x time interaction *F*_(2,26)_=0.25, p = 0.78; group (opsin) effect *F*_(1,13)_=0.097, p = 0.76; time effect *F*_(1.78,23.2)_=0.49, p = 0.60. Post-hoc ChRmine vs Control: 0–3 min p = 0.99, 3–6 min p = 0.99, 6–9 min p = 0.99). **t**, Average velocity (cm/s) of mice in the OFT test for control (grey) and ChRmine (red) mice subjected to chronic optical stimulation (n = 6 (control) and 9 (ChRmine) mice, two-way repeated-measures ANOVA with Bonferroni post-hoc test: group (opsin) x time interaction *F*_(2,26)_=0.29, p = 0.78; group (opsin) effect *F*_(1,13)_=0.005, p = 0.94; time effect *F*_(1.34,17.4)_=17.3, p = 2.6e-4. Post-hoc ChRmine vs Control: 0–3 min p = 0.99, 3–6 min p = 0.99, 6–9 min p = 0.99). **u**, Total distance travelled (cm) mice during the OFT test for control (grey) and ChRmine (red) mice subjected to chronic optical stimulation (n = 6 (control) and 9 (ChRmine) mice, two-way repeated-measures ANOVA with Bonferroni post-hoc test: group (opsin) x time interaction *F*_(2,26)_=0.29, p = 0.75; group (opsin) effect *F*_(1,13)_=0.005, p = 0.94; time effect *F*_(1.34,17.4)_=17.3, p = 2.6e-4. Post-hoc ChRmine vs Control: 0–3 min p = 0.99, 3–6 min p = 0.99, 6–9 min p = 0.99). Data represent mean ± s.e.m.

To test whether heart rhythms directly set by this optical pacemaker could influence behaviour, we optogenetically induced intermittent ventricular tachycardia (900 bpm for 500 ms every 1,500 ms) to mimic non-sustained arrhythmias that are observed during stressful contexts^34–36^, while shortening the duration of decreases in systolic blood pressure and avoiding incidental heating from light-delivery devices (Fig. 2b and Extended Data Figs. 3j–m and 4c). We first assessed the appetitive or aversive effects of optical pacing using a real-time place-preference (RTPP) assay (Fig. 2c). Mice spent an equal proportion of time on the paced and non-paced sides of the two-chamber arena, and showed no difference in locomotion compared to littermate controls—revealing that optically induced intermittent tachycardia was not intrinsically aversive and did not cause locomotor impairment (Fig. 2d,e). Optical pacing also did not modulate pain perception during a hot-plate test, with paced mice exhibiting comparable behavioural responses to control mice (Extended Data Fig. 5a–c).

## Anxiety-like state evoked by cardiac pacing

By contrast, when we tested for anxiety-related behaviour using an elevated plus maze (EPM) assay, the same mice exhibited limited exploration of the open (exposed) arms of the apparatus after optical pacing, compared to control mice, preferring to remain within the protected areas of the closed arms (Fig. 2f,g). Paced mice also avoided the centre area during an open field test (OFT) (Fig. 2h,i). We observed no effects from illumination alone in control (opsin-negative) mice, and baseline anxiety levels between control and virally transduced groups were similar (Extended Data Fig. 5d–h). Increased anxiety-like behaviour induced by optical pacing during the EPM and OFT assays was similarly observed in female cohorts (Extended Data Fig. 5i–l). We found that mice that received intermittent cardiac pacing within baseline ranges (660 bpm) rather than elevated (900 bpm) ranges did not exhibit behavioural differences compared to control mice during the EPM or OFT (Extended Data Fig. 5m–p). Because continuous ventricular pacing can have a long-lasting effect on animal health^20,21^, we also assessed for potential changes in baseline anxiety levels and mobility in mice that were subjected to longer-term treatments of intermittent tachycardia (one-hour sessions every other day for two weeks) and did not observe locomotor or behavioural differences in these mice when compared to control mice during the EPM and OFT (Extended Data Fig. 5q–u).

**Extended Data Fig. 6:**
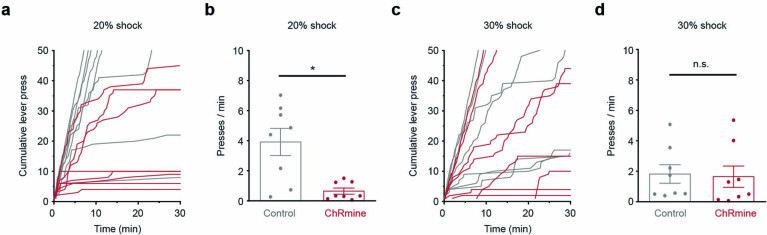
Operant lever-pressing task at higher risk of shock. **a**, Cumulative lever presses during 20% shock session for control (grey) and ChRmine-expressing (red) mice (n = 8 mice per group). Experiment was performed otherwise identically to the behaviour described in Fig. 2. **b**, Lever-pressing rate averaged across the entire 20% shock trial session (n = 8 mice per group, Two-tailed *t*-test, *p = 0.0367). **c**, Cumulative lever presses during 30% shock session. **d**, Lever-pressing rate averaged across the entire 30% shock trial session (n = 8 mice per group, Two-tailed t-test, p = 0.7663). Data represent mean ± s.e.m.

We further investigated whether this context-dependent enhancement of anxiety-related behaviour could translate to a classical operant task, by using a trial-based variation of the Vogel conflict task in which water-restricted mice show willingness to seek a water reward even when the reward is coupled to a risk of mild shock^37^ (Fig. 2j). Mice that received cardiac pacing performed similarly to control littermates when allowed to freely press for water with no delivery of the aversive stimulus (Fig. 2k–n). However, when random shocks were introduced in 10% of trials, optically paced mice were found to suppress or terminate water-seeking altogether (Fig. 2l,m). These mice exhibited increased apprehension, as revealed both by an overall decreased lever-pressing rate and by an increased time to the next subsequent lever press after a shock trial—consistent with the heightened levels of anxiety that were observed during the EPM and OFT (Fig. 2m,n). By contrast, control mice exhibited reduced water-seeking only when the frequency of shock trials was increased to 30% (Extended Data Fig. 6). This context-dependent influence of cardiac pacing on anxiety-like behaviour suggested that higher-order brain function was involved in the processing of interoceptive cues.

## Optical pacing increases insula activity

**Fig. 3:**
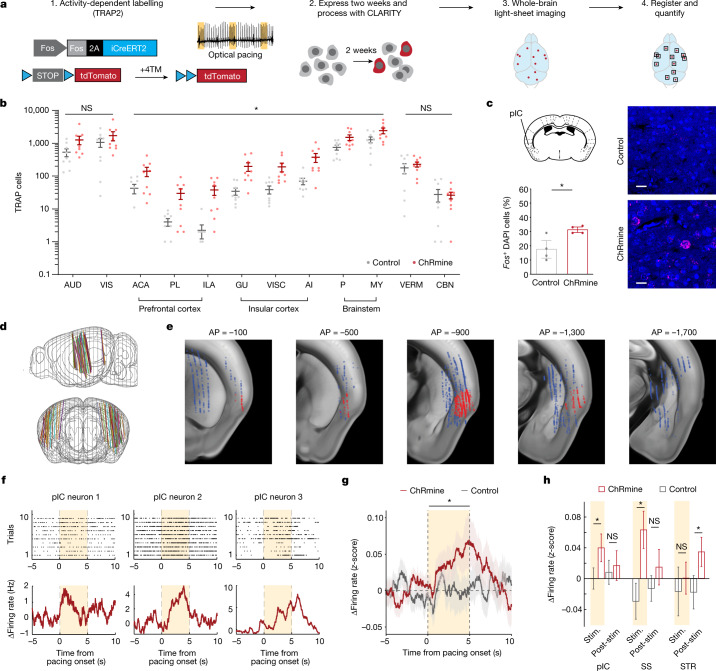
Whole-brain screen for regions that are activated by optically induced tachycardia. **a**, Schematic for whole-brain activity mapping to identify regions that are activated by optically paced tachycardia. Double-transgenic TRAP2;Ai14 reporter mice were injected with 4-hydroxytamoxifen (4TM) and treated with optically induced tachycardia for 15 min. After two weeks of tdTomato reporter gene expression, mice were euthanized and processed with CLARITY. Whole brains were imaged with a light-sheet microscope, followed by automated registration to a common brain atlas, cell segmentation and quantification to identify brain regions with differential accumulation of activated TRAP (tdTomato^+^) cells. **b**, Regional cell counts of paced (ChRmine, red) versus control (grey) cohorts sorted from anterior to posterior anatomical regions with select regions from the central autonomic network with increased TRAP cells and regions outside of the central autonomic network without statistical significance (*n* = 9 per group; multiple two-sided *t*-tests corrected for multiple comparisons with the Benjamini and Hochberg method (*false discovery rate (FDR) = 10%)). Significantly activated regions include the prefrontal cortex (ACA, PL and ILA), insular cortex (GUI, VISC and AI) and brainstem (P and MY). Non-significant regions include primary sensory cortices (AUD, VIS) and the cerebellum (VERM, CBN). **c**, Percentage of cells that are *Fos*^+^, determined from in situ hybridization for *Fos* mRNA (magenta) after cardiac pacing in control and ChRmine-expressing mice in the pIC (*n* = 4 mice per group; unpaired two-tailed *t*-test, *P = 0.020). Scale bars, 20 µm. **d**, Electrode tracks from *n* = 5 mice (3 ChRmine and 2 control) over 60 recording sessions co-registered to the common Allen Brain Atlas. **e**, Locations of recorded single units overlaid onto the Allen Brain Atlas. Red denotes units in the insular cortex. AP, anterior–posterior. **f**, Spike raster and changes in firing rate for three example insular neurons after 900-bpm pacing. **g**, Population-averaged changes in firing rate of insular neurons from ChRmine (red, *n* = 391) or control (grey, *n* = 228) mice (one-sided *P* values from hierarchical bootstrap: *P* = 0.026 (during 5 s pacing); *P* = 0.357 (during 5 s after pacing)). **h**, Average change in baseline firing rate per brain region across 5-s epochs during and after photostimulation in control (grey) and ChRmine-expressing (red) mice. Single units were obtained from the pIC (*n* = 228 (control), *n* = 391 (ChRmine)); somatosensory cortex (SS; *n* = 77 (control), *n* = 368 (ChRmine)); and striatum (STR; *n* = 70 (control), *n* = 800 (ChRmine)). One-sided *P* values from hierarchical bootstrap: pIC: *P* = 0.026 (stimulation; stim.), *P* = 0.36 (post-stimulation; post-stim.); SS: *P* = 0.0014 (stim.), *P* = 0.16 (post-stim.); STR: *P* = 0.29 (stim.), *P* = 0.036 (post-stim.). Data are mean ± s.e.m.

**Extended Data Fig. 7:**
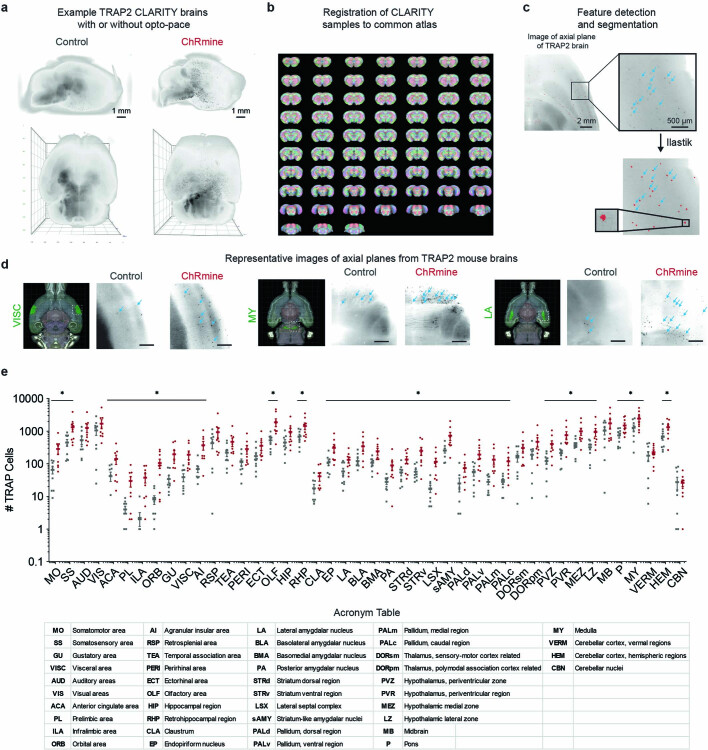
Generation of brain-wide activity maps during optical pacing. **a**, Representative whole-brain CLARITY sagittal (top) and axial (bottom) images of control and ChRmine expressing mice exposed to optical pacing. Heart-targeted ChRmine expression resulted in increased tdTomato+ cells throughout the brain (seen as black dots) relative to control. **b**, Following light-sheet imaging, each image stack was registered to a common Allen Reference Atlas using our previously reported computational pipeline^40^. Depicted are coronal slices across the brain with the overlaid anatomical atlas, where different anatomical regions are depicted with different colours. **c**, Representative raw image of single plane of TRAP2-tdTomato brain imaged under light-sheet microscope (left), and after detection by a supervised classifier (Ilastik, Arivis plugin), where single-cells are outlined in red (right, bottom). Blue arrows indicate example features that were detected by the classifier. **d**, Example CLARITY light-sheet images of control and ChRmine-paced mice in the visceral cortex (VISC), Medulla-behavioural state related (MY), and in the lateral amygdalar nucleus (LA), which are outlined in green in the axial reference slice. Scale bar=500 µm. **e**, Regional cell counts of paced (ChRmine, red) vs control (grey) cohorts sorted from anterior to posterior in all anatomical regions (n = 9 per group, multiple two-sided t-tests corrected for multiple comparisons with the Benjamini and Hochberg method (*FDR=10%). p-values: MO p = 0.025, SS p = 0.038, AUD p = 0.082, VIS p = 0.31, ACA p = 0.050, PL p = 0.027, ILA p = 0.0086, ORB p = 0.012, GU p = 0.013, VISC p = 0.018, AI p = 0.016, RSP p = 0.20, TEA p = 0.11, PERI p = 0.074, ECT p = 0.10, OLF p = 0.017, HIP p = 0.10, RHP p = 0.052, CLA p = 0.092, EP p = 0.035, LA p = 0.028, BLA p = 0.051, BMA p = 0.036, PA p = 0.043, STRd p = 0.029, STRv p = 0.015, LSX p = 0.012, sAMY p = 0.037, PALd p = 0.036, PALv p = 0.033, PALm p = 0.024, PALc p = 0.012, DORsm p = 0.16, DORpm p = 0.095, PVZ p = 0.016, PVR p = 0.020, MEZ p = 0.029, LZ p = 0.033, MB p = 0.28, P p = 0.030, MY p = 0.054, VERM p = 0.50, HEM p = 0.043, CBN p = 0.92). Acronyms for each major brain region is listed.

**Extended Data Fig. 8:**
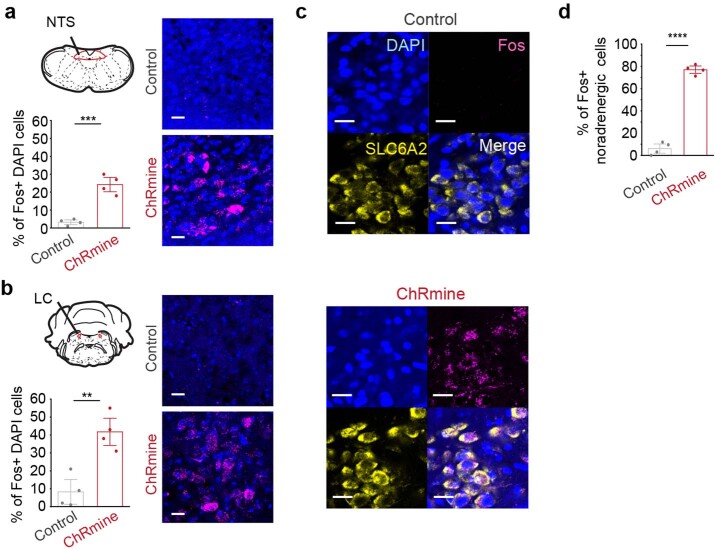
Optically induced tachycardia increases *Fos* mRNA expression in brain regions associated with the central autonomic network. **a**,**b**, Percentage of cells that are *Fos*^+^ determined from in situ hybridization for *Fos* mRNA (magenta) following cardiac pacing in control and ChRmine-expressing mice in the nucleus of the solitary tract (NTS) (**a**) and in the locus coeruleus (LC) (**b**) (n = 4 mice per group, unpaired two-tailed t-test, ***p = 0.00029 (NTS), **p = 0.0027). We have also performed in situ hybridization for *Fos* mRNA in the nodose ganglion and observed potential induction in these vagal sensory neurons by the cardiac signals (n = 4, control 0.96% ± 0.7; ChRmine 5.29% ± 1.6, unpaired two-tailed t-test, p = 0.04). **c**, Confocal images of the locus coeruleus stained for *Fos* (magenta), the norepinephrine transporter *Slc6a2* (yellow) and DAPI (blue). **d**, Quantification of neurons that express *Slc6a2* that also co-localize with *Fos* during optical pacing (n = 4 mice per group, unpaired two-tailed t-test: ****p = 1.0e-7). Scale bar=20 µm. Data represent mean ± s.e.m.

We therefore next used the optical pacemaker to identify potential neural correlates and mechanisms of this observed behaviour along the heart–brain axis. First, transgenic TRAP2 mice, in which neurons with increased expression of the immediate early gene *Fos* can be labelled with tdTomato as a marker for neural activation^38^, were used to perform a brain-wide screen to identify regions that were affected by optical pacing (Fig. 3a,b and Extended Data Fig. 7). Whole-brain tissue clearing^39^, registration to a reference brain atlas^40^ and automated cell counting together revealed that a number of brain regions exhibited increased expression of tdTomato in optically paced mice. These included areas associated with the central autonomic network^41,42^, such as the insular cortex (including its visceral area (VISC), gustatory area (GU) and agranular insular area (AI)), prefrontal cortex (including the infralimbic area (ILA), prelimbic area (PL) and anterior cingulate area (ACA)) and brainstem (including the pons (P) and medulla (MY)) (Fig. 3b). Meanwhile, pointing to specificity, many other cortical regions that are not known to be involved in autonomic or interoceptive processing were not significantly activated, including primary sensory auditory (AUD) and visual (VIS) cortical areas, as well as the cerebellar vermis (VERM) and cerebellar nuclei (CBN) (Fig. 3b). Consistent with the TRAP2 mapping results, optical pacing similarly increased the endogenous expression of *Fos* mRNA in the posterior insular cortex (pIC) and in the brainstem (Fig. 3c and Extended Data Fig. 8). In particular, sensory relay circuits of the nucleus tractus solitarius (NTS), as well as noradrenergic neurons in the locus coeruleus (LC), which are involved in arousal and stress^43^, also exhibited prominent *Fos* labelling (Extended Data Fig. 8).

We next investigated the cardiac-pacing-induced neural dynamics at single-neuron resolution using in vivo electrophysiology in awake mice (Fig. 3d–h). On the basis of the observed increased *Fos* expression in the pIC after cardiac pacing, and because the insular cortex is a key cortical hub for interoception^44^, we used four-shank Neuropixels 2.0 probes to obtain rich multi-regional recordings of the pIC and surrounding regions (Fig. 3d,e). Notably, we observed pacing-evoked increases in insular activity at both the single-unit and population levels (Fig. 3f,g), whereby pIC neurons were acutely activated by cardiac stimulation with heterogeneous temporal dynamics and returned to basal levels of activity after pacing offset. By contrast, no significant changes in activity were recorded in control mice during photostimulation, ruling out potential light- or heat-induced representations in this region (Fig. 3g). We also observed a substantial diversity in temporal dynamics triggered by pacing in other recorded regions, including acute responses in the somatosensory cortex and delayed responses in the striatum during stimulation offset (Fig. 3h). Our data show that the pIC and other regions of the central autonomic network are distinctly engaged by optically evoked tachycardia^41,42^, and are in line with human neuroimaging studies that have correlated these brain areas with cardiac interoception^45–47^.

## Insular inhibition reduces cardiac anxiety effects

**Fig. 4:**
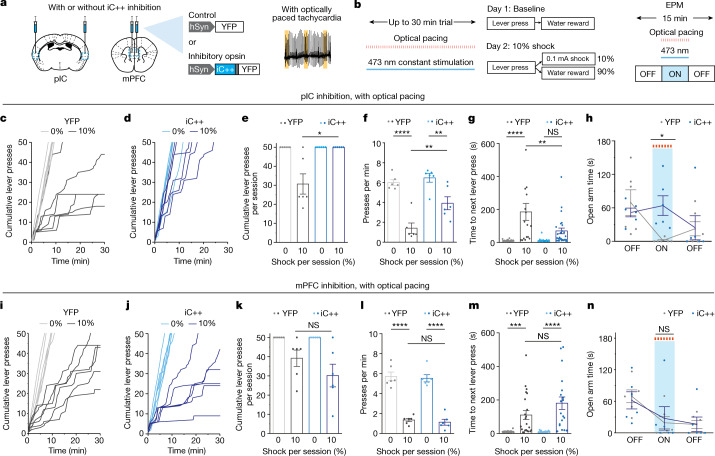
Optogenetic inhibition of the posterior insula attenuates the anxiogenic response from optical pacing. **a**, Illustration of the experimental protocol for simultaneous optically induced tachycardia and optogenetic inhibition of the pIC or mPFC using AAVdj-hSyn::iC++-YFP or control virus (YFP only). **b**, Left, conditions for the Vogel conflict task, in which mice received both 473-nm constant illumination in the pIC or mPFC and optically induced tachycardia during the behavioural task. Right, illustration of the experimental protocol for simultaneous optogenetic inhibition of the pIC with optical pacing during the EPM test. **c**,**d**, Cumulative lever presses during baseline (day 1) and 10% shock (day 2) sessions for mice expressing control (YFP) (**c**) or iC++ (**d**) in the pIC with optical pacing (*n* = 6 mice per group). **e**, Cumulative number of lever presses completed in each session. Owing to increased apprehension, only 1 out of 6 control mice completed the 50-lever-press session on the 10% shock session (*n* = 6 per group; two-sided Wilcoxon rank-sum test, *P = 0.0152). **f**, Average lever-pressing rate for 0% and 10% shock experimental sessions. Note that iC++ inhibition partially restores overall rates of lever pressing, but not to baseline levels (*n* = 6 per group; two-way ANOVA with Bonferroni post hoc test: group × condition interaction *F*_(1,10)_ = 5.533, *P* = 0.0405; group (opsin) effect *F*_(1,10)_ = 7.439, *P* = 0.0213; condition (shock) effect *F*_(1,10)_ = 67.8, *P* < 0.0001. Bonferroni post hoc: 0% shock YFP versus iC++, *P* > 0.9999; 10% shock YFP versus iC++, **P = 0.0036; YFP 0% versus 10% shock, ****P < 0.0001; iC++ 0% versus 10% shock, **P = 0.0039). **g**, Time to next lever press after shock. Note that iC++ inhibition reduces apprehension to no-shock levels (*n* = 30, 17, 30 and 30 presses per group in YFP 0%, YFP 10%, iC++ 0% and iC++ 10%; two-way ANOVA with Bonferroni post hoc test: group (opsin) × condition (shock) interaction *F*_(1,103)_ = 8.7, *P* = 0.0039; group (opsin) effect *F*_(1,103)_ = 8.6, *P* = 0.0041; condition (shock) effect *F*_(1,103)_ = 35.6, *P* < 0.0001. Bonferroni post hoc: YFP 0% versus 10%, ****P < 0.0001; iC++ 0% versus 10%, *P* = 0.099; 0% YFP versus iC++, *P* > 0.9999; 10% YFP versus iC++, **P = 0.0011). **h**, Time spent in open arms during 5-min epochs of EPM exploration (*n* = 6 per group; two-way repeated-measures ANOVA with Bonferroni post hoc test: group (opsin) × time interaction *F*_(2,20)_ = 3.543, *P* = 0.0482; group (opsin) effect *F*_(1,10)_ = 1.251, *P* = 0.2894; time effect *F*_(2,20)_ = 3.058, *P* = 0.0694. Bonferroni post hoc: ON epoch YFP versus iC++, *P = 0.0323). **i**,**j**, Cumulative lever presses with the same conditions as **c**,**d** but with expression of YFP (**i**) or iC++ (**j**) in the mPFC (*n* = 6 mice). **k**, Cumulative number of lever presses during each session. Note that in 10% shock sessions, both YFP- and iC++-expressing mPFC mice cease lever pressing (*n* = 6 per group; two-sided Wilcoxon rank-sum test, *P* = 0.2987). **l**, Average lever-pressing rate for 0% and 10% shock sessions (*n* = 6 per group; two-way repeated-measures ANOVA with Bonferroni post hoc test: group × condition interaction *F*_(1,10)_ = 0.002521, *P* = 0.9609; group (opsin) effect *F*_(1,10)_ = 0.4370, *P* = 0.5235; condition (shock) effect *F*_(1,10)_ = 154.1, *P* < 0.0001. Bonferroni post hoc: 0% shock YFP versus iC++, *P* > 0.9999; 10% shock YFP versus iC++, *P* > 0.9999; YFP 0% versus 10% shock, ****P = 1.1 × 10^−5^; iC++ 0% versus 10% shock, ****P = 1.0 × 10^−6^). **m**, Time to next lever press after shock (*n* = 30, 22, 30 and 19 presses per group in mPFC YFP 0%, YFP 10%, iC++ 0% and iC++ 10%; two-way ANOVA with Bonferroni post hoc test: group (opsin) × condition (shock) interaction *F*_(1,97)_ = 3.703, *P* = 0.05725; group (opsin) effect *F*_(1,97)_ = 3.610, *P* = 0.0604; condition (shock) effect *F*_(1,97)_ = 54.18, *P* < 0.000001. Bonferroni post hoc: YFP 0% versus 10%, ***P = 0.0009; iC++ 0% versus 10%, ****P = 2.95 × 10^−8^; 0% YFP versus iC++, *P* > 0.9999; 10% YFP versus iC++, *P* = 0.0698). **n**, Time spent in open arms during 5-min epochs of EPM exploration with (iC++, blue) and without (YFP, grey) mPFC inhibition (*n* = 6 per group; two-way repeated-measures ANOVA with Bonferroni post hoc test: group (opsin) × time interaction *F*_(2,20)_ = 0.3929, *P* = 0.6802; group (opsin) effect *F*_(1,10)_ = 0.00039, *P* = 0.9846; time effect *F*_(2,20)_ = 17.41, *P* < 0.0001. Bonferroni post hoc: ON epoch YFP versus iC++, *P* > 0.9999).

To determine whether the anxiogenic circuitry recruited by cardiac pacing could be specifically modulated to influence behaviour, we next performed optogenetic inhibition with the 473 nm (blue light)-activated inhibitory channelrhodopsin iC++. We targeted the pIC (with well-established roles in both processing and regulating cardiac sensory signals and anxiety-related behaviours^45–48^) and the medial prefrontal cortex (mPFC) (crucially involved in reward and aversion processing, as well as associated with cardiovascular arousal^49^). To perform simultaneous optogenetic inhibition of the cortex and optically paced tachycardia, we bilaterally injected AAVdj-hSyn::iC++-eYFP or AAVdj-hSyn::eYFP control virus and implanted fibre-optic cannulas into the pIC or mPFC of mice expressing ChRmine in the heart^50^ (Fig. 4a). As expected, in eYFP-expressing control mice, we found that 473-nm illumination of the pIC did not affect the reduction of water-seeking by optical pacing in trials with a risk of shock (Fig. 4b,c). By contrast, the same intervention in mice expressing iC++ instead of eYFP alone in pIC did reverse the reduction of water-seeking (Fig. 4d–g); all mice receiving pIC inhibition completed the water-retrieving task and exhibited decreased apprehension, with the time to next lever press after shock reduced to near baseline levels (Fig. 4g). Similarly, iC++ inhibition increased open-arm exploration time during the EPM assay in optically paced mice relative to eYFP controls (Fig. 4h).

**Extended Data Fig. 9:**
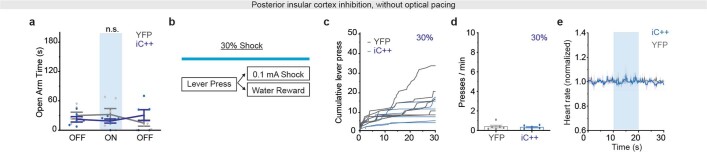
Behavioural and physiological effects from inhibition of the posterior insula. **a**, Mice were evaluated on the EPM with iC++ inhibition only during the “ON” epoch, with no optical pacing vest (n = 6 mice per group; two-way repeated-measures ANOVA: group (opsin) x time interaction *F*_(2,20)_=2.569, p = 0.1016; group (opsin) effect *F*_(1,10)_=0.0392, p = 0.8470; time effect *F*_(2,20)_=0.1486, p = 0.8629). Note inhibition of pIC did not significantly alter time spent in the open arms of the EPM. **b**, Schematic overview of the pIC inhibition experiment. Mice were evaluated on the Vogel conflict task with elevated chance of shock (30%) while wearing the optical pacing vest but without receiving cardiac stimulation. Optogenetic inhibition was performed during the entire duration of the 30 min trial. **c**, Individual traces of lever presses during shock day with 30% chance of shock and bilateral pIC inhibition (n = 6 mice per group). **d**, Lever-pressing rate averaged across entire 30% shock session (n = 6 mice per group). **e**, Heart rate as a function of time during pIC inhibition (blue) (n = 3 mice per group). Data represent mean ± s.e.m.

The attenuation of the anxiogenic effect of optical pacing exhibited specificity to pIC inhibition; inhibition of the mPFC did not decrease cardiac-associated anxiogenic behaviours relative to eYFP controls (Fig. 4i–n). To test for any direct anxiolysis from optogenetic inhibition of the pIC (that is, not through modulation of the cardiac-pacing effect), we performed the EPM assay and the lever-pressing task in the absence of cardiac pacing and with 30% shock trials to allow the detection of pacing-independent apprehensive behaviour (Extended Data Fig. 9a–d). Inhibition of the pIC without cardiac pacing did not increase open-arm exploration, affect lever-press suppression or influence heart rate (Extended Data Fig. 9). Thus, pIC inhibition alone appeared to be insufficient to induce anxiolysis, consistent with previous reports^48^. Together, these results are in line with a model in which the pIC is important for mediating the anxiety-related and apprehensive behaviours that arise from direct cardiac pacing.

## Discussion

In this study, we have developed a method for noninvasive optogenetic control of specific cardiac rhythms during active behaviour. We show that the optically induced tachycardia was not intrinsically aversive, but rather elicited anxiety-like behaviours and apprehension in potentially risky environments. Although diverse mechanisms may contribute to this effect, we consider that anxiogenic effects of evoked tachycardia are not likely to be mediated through a reduction in blood pressure^51^, as drugs that reduce systolic blood pressure tend to be anxiolytic (for example, propranolol and clonidine) or neutral (for example, Ca^2+^-channel blockers). Our observation of anxiogenesis in response to increased heart rates (900 bpm or 15 Hz) is in line with clinical observations that accelerated heart rates—but not other forms of altered haemodynamics (for example, increased heart rate variability)—are associated with panic and other anxiety-related disorders^52,53^. The altered rate, rather than the external nature of cardiac contraction timing, appears to be important; for example, we found that intermittent or asynchronous stimulation close to baseline heart rates at 660 bpm (11 Hz) did not result in anxiety-like behaviour.

In further investigations of the mechanisms that underlie these behaviours, we found that optogenetic pacing activated the pIC, consistent with studies of cardiovascular control in anaesthetized rodents^54^ and neuroimaging studies of cardiac interoception and reflex control in humans^45–47^, including in the setting of panic and anxiety^49,55^. It remains unclear whether this pathway can be modulated by peripheral baroreceptive sensory neurons or other sensory mechanisms that detect changes in blood pressure^56–58^, and it is possible that there are additional ways in which cardiac viscerosensory information can be relayed to higher cortical areas^41,42^. The anxiogenic behavioural effects of cardiac pacing were attenuated during optogenetic inhibition of the pIC, suggesting that the insula has a causal role in integrating sensory information from the heart with a contextual assessment of environmental risk to produce adaptive behavioural patterns. Our findings support the idea that the insular cortex is involved in monitoring not only consummatory^59–61^ but also entirely internal interoceptive states to instruct relevant behavioural responses, as predicted from human neuroimaging studies of cardiac interoception^45–47^.

This study shows that cell-type-specific, temporally precise, noninvasive perturbation of organ-scale physiology is possible in fully intact, freely behaving mammals. Although we have applied our approach mainly to study animal behaviour over a period of minutes, future integration with miniaturized wireless devices^15^ may facilitate longer-term studies to modulate targeted populations of cells over days to weeks while alleviating the need for intimate contact with the light source. Furthermore, refinement in cell-type-targeting strategies may enable minimally invasive to noninvasive optogenetic dissection of specific cell types (for example, pacemaker cells^28^, Purkinje fibres^27^ and cardiac ganglions^12^) to determine their effects on regulating cardiac electrophysiology and behaviour. Finally, our approach, which requires no specialized optoelectronics or surgery, has the potential for broad application to a range of physiological systems throughout the body—opening up numerous opportunities to explore the complex interactions between physiological systems in health, disease and treatment.

## Methods

### Mice

All animal procedures followed animal care guidelines approved by Stanford University’s Administrative Panel on Laboratory Animal Care (APLAC) and guidelines of the National Institutes of Health. Investigators were not blinded to the genotypes of the mice. Male and female wild-type C57BL6/J (JAX 0064) mice were used for most behavioural experiments unless specified otherwise, and all mice were 8–12 weeks old at the time of starting behavioural experiments. Mice were housed in plastic cages with disposable bedding on a standard light cycle with food and water available ad libitum, except when placed on water restriction. When on water restriction, mice were provided with 1 ml of water each day and maintained above 85% of baseline weight. Behavioural experiments were performed during the dark phase.

### Molecular cloning

A 685-bp fragment containing the promoter region of the mouse troponin gene was amplified from a wild-type mouse using CGCACGCGTGAGGCCATTTGGCTCATGAGAAGC and CATGGATCCTCTAGAAAGGGCCATGGATTTCCTG primers, cloned upstream of ChRmine-p2A-oScarlet using MluI and BamHI sites in an AAV backbone, sequence-verified and tested for expression in dissociated neonatal cardiomyocytes.

### In vitro cardiomyocyte experiments

Dissociated neonatal mouse cardiomyocytes prepared using the Pierce Isolation Kit (Thermo Fisher Scientific, 88289) were transfected with rAAV-mTNT::ChRmine-p2A-oScarlet (1 µl of 8 × 12 viral genomes (vg) per ml in 500 µl of medium). Three to five days after infection, individual cardiomyocytes were identified under a light microscope. Optical stimulation was provided by a Spectra X Light engine at 585 nm (LumenCore) coupled to a Leica DM LFSA microscope and synchronized with video recording at 100 fps using LabView software. Laser power leaving the imaging objective was measured with an optical power meter (Thorlabs PM100D). Videos were analysed for contraction using custom scripts in MATLAB.

### In vivo systemic viral delivery

Wild-type mice aged three to four weeks were anaesthetized with isoflurane and rAAV-mTNT::ChRmine-p2A-oScarlet (2 ×10^11^ vg per mouse) or vehicle was delivered by retro-orbital injection. Our selected titres were previously used for systemic viral transduction of ChR2 in the heart^26^. A total volume of 60 µl 0.9% NaCl saline solution was injected into the right retro-orbital sinus using a 28G needle, then allowed to recover on a warming pad before being returned to the home cage.

### Optical pacemaker in vivo characterization

Mice were tested three weeks after injection of the pacemaker virus. Electrocardiography signals were collected using commercial instruments (Rodent Surgical Monitor+, Indus Instruments), with anaesthetized mice placed in a supine position and limbs placed in contact with electrode pads via a conductive gel. A 594-nm laser (LaserGlow) was attached to a fibre-optic patch cord (Thorlabs) terminating in a 200-µm-diameter, 0.39-NA fibre (Thorlabs) which was positioned against the chest. Optical power was adjusted using the laser’s built-in power modulator and measured with an optical power meter (Thorlabs) at the fibre tip. Stimulation was performed with a pulse width of 10 ms and an inter-pulse interval ranging from 120 ms (equivalent to 500 bpm) to 67 ms (900 bpm), controlled by a TTL signal generator (Master-8). Heart rate (bpm) was derived from the heart rate interval between successive R waves (RR interval) obtained from ECG recordings. Fidelity of photoactivated QRS complexes was quantified by counting the number of beats at a set frequency divided by the number of total beats measured during the middle 20 s of a 30-s stimulation period.

### Measurements of systolic blood pressure

Mice were anaesthetized (1.5–2% isofluorane) and placed in the supine position with the chest shaved. Systolic blood pressure measurements were performed using a 1.4-F pressure sensor mounted Millar catheter (SPR-671, ADInstruments) and recorded using LabChart 7 Pro (ADInstruments). The catheter was inserted via the right carotid artery into the left ventricle. A 589-nm laser was used to deliver 240 mW mm^−2^ light across intact chest at either constant or intermittent (500 ms ON, 1,500 ms OFF) optical stimulation at 900 bpm with a 10-ms pulse width for 30 s to assess optogenetic pacing effects on systolic blood pressure in real time.

### Wearable optical pacemaker hardware

Custom-made wearable optical stimulators were constructed using 3 × 4.5 mm 591-nm PC Amber Rebel LEDs (Luxeon LXM2-PL01-0000). 30AWG flexible silicone wire (Striveday) was soldered to the LED pad and coated with electrically insulating, thermally conductive epoxy (Arctic Alumina), and adhered to copper sheet cut to 10 × 15 mm for thermal dissipation and subsequently glued to a fabric vest designed for freely moving mouse behaviour (Coulbourn A71-21M25). Wiring was held in place on the vest using hot glue and the free ends were inserted into a breadboard for stimulus control by an LED Driver (Thorlabs LEDD1B T-Cube). The optical power was set to 160–240 mW mm^−2^ measured from the surface of the LED. Light was delivered at intervals consisting of a 10-ms pulse width at 15 Hz (900 bpm) for 500 ms with 1,500 ms OFF time by using either a Master-8 or an Arduino microcontroller synchronized to behaviour recording software. Computer-aided design schematics were created with Onshape. Thermal measurements were performed using a FLIR C2 Compact thermal camera (FLIR) and the thermal profile at the surface of the micro-LED is plotted in Extended Data Fig. 4c.

### Freely moving behaviour with pacemaker

All mice were habituated to the experimenter and handled for at least three days, and in addition allowed to acclimatize to wearing optical pacemaker hardware for at least five days, before behavioural experiments. Fur over the chest was removed (Nair) at least five days before behavioural experiments. Mice were briefly anaesthetized with isoflurane before the placement of the optical pacing vest and allowed to fully recover in the home cage (at least 1 h) before experiments. We used a stimulation protocol consisting of a 10-ms pulse width at 15 Hz (900 bpm) with 500 ms ON time and 1,500 ms OFF time to introduce intermittent tachycardia or 10-ms pulse width at 11 Hz (660 bpm) with a Poisson distribution to introduce increased heart rate variability. Mice received optical stimulation during the ON periods of the behavioural assay from the wearable micro-LED device in both control and ChRmine cohorts. No statistical difference in behaviour was observed between virally transduced and control groups at baseline, suggesting that there were no side effects from transgene delivery. No statistical difference in behaviour was observed in control groups before, during and after optical stimulation, suggesting that there were no effects from light delivery alone.

### RTPP

Mice were placed in a custom-built RTPP chamber (30.5 × 70 cm) on day 1 to determine their baseline preference for each side of the chamber. Behavioural tracking was performed using blinded automated software (Noldus Ethovision). On day 2, mice were stimulated whenever they were on one side of the chamber. Stimulation sides were randomly assigned and counterbalanced across mice. Each session lasted 20 min.

### EPM

The EPM was made of grey plastic (Med Associates). Mice were gently placed in the closed arm of the EPM. Mice were allowed to freely explore the maze for a 5-min baseline ‘off’ period, followed by a 5-min ‘on’ period during which optical stimulation was delivered, and finally a 5-min ‘off’ period. Behavioural tracking was performed using blinded automated software (Noldus Ethovision) and the overall time spent in open arms was reported for each epoch.

### OFT

Mice were placed in a 60 × 60-cm arena and allowed to freely explore during a 9-min session. Optical stimulation was delivered during the middle 3-min epoch. Movement was tracked with a video camera positioned above the arena. To assess anxiety-related behaviour, the chamber was divided into a peripheral and centre (48 × 48 cm) region.

### Operant lever-pressing task

Water-restricted mice were trained to lever press for a small water reward (around 10 μl water) while freely moving in an operant condition box containing a single retractable lever and a shock grid floor (Coulbourn). Mice were allowed to retrieve a maximum of 50 rewards per day, and sessions were terminated after all rewards had been retrieved or after 30 min. After each lever press, the lever was retracted for 5 s before extending again. After mice retrieved 50 rewards for at least 3 consecutive days (typically 2–3 weeks of training), they were allowed to proceed with stimulation experiments. On shock days, mice were given a 1-s, 0.1-mA foot shock after 10% of lever presses instead of water. Shocks were delivered in a pseudorandom order on lever-press trials 5, 13, 24, 31 and 44, and the time to the next lever press was measured from the time elapsed for these trials until the subsequent lever press. During stimulation experiments (both baseline and shock days), optical stimulation was delivered throughout the experiment. Water was delivered using a custom set-up consisting of a lick spout (Popper and Sons, stainless steel 18-gauge) and a solenoid (Valcor, SV74P61T1) controlled by a microcontroller (Arduino Uno R3). Licking was monitored using a capacitive sensing board (Arduino Tinker Kit) wired to the lick spout and interfacing with the microcontroller. Shocks were delivered using an 8-pole scrambled shock floor (Coulbourn). Behavioural stimuli—lever presentations and retractions, and shocks—were controlled with Coulbourn Graphic State software. The timing of lever presses and licks was also recorded at 5 kHz using a data-acquisition hardware (National Instruments, NI PCIe-6343-X).

### TRAP2 labelling

*Fos*^*2A-iCreER*^ (TRAP2; JAX 030323) mice were backcrossed onto a C57BL6/J background and bred with *B6;129S6-Gt(ROSA)26Sor*^*tm14(CAG-tdTomato)/Hze*^*/J* (Ai14; JAX 007908) mice, as previously described^38^. Both male and female mice were used for TRAP2 labelling experiments. Mice were injected retro-orbitally with rAAV9-mTNT-ChRmine-oScarlet or a vehicle control at three to four weeks of age. Four weeks later, mice were handled and acclimatized to fresh clean cages and optical pacing equipment for at least seven days before labelling. On the day of labelling, mice were allowed to acclimatize to optical pacing equipment for at least 2 h in a fresh clean cage with food and water, stimulated for 15 min (10-ms pulse width at 15 Hz for 500 ms every 1,500 ms) and left undisturbed for 2 h, at which time they were injected intraperitoneally with 5 mg kg^−1^ 4-hydroxytamoxifen (Sigma) dissolved in normal saline containing 1% Tween-80 and 2.5% DMSO (as described previously^37,39^). Mice were then returned to their home cage and were euthanized at least two weeks later to allow for full expression of the fluorophore.

### Whole-brain CLARITY and analysis

Mice were perfused with ice-cold phosphate-buffered saline (PBS) and 4% paraformaldehyde (PFA), then post-fixed in a 1% CLARITY hydrogel solution (1% acrylamide, 0.003125% bis-acrylamide, 4% PFA and 0.25% VA-044 in 1× PBS) for 2 days. Tissue was degassed, polymerized at 37 °C for 4 h and washed with 200 mM sodium borate with 4% sodium dodecyl sulfate solution overnight. Tissue was then electrophoretically cleared for 3–7 days at 80 V (Life Canvas), passively cleared for an additional 2 days, then washed in PBS containing 0.2% Triton-X and 0.02% sodium azide at least 6 times at 37 °C. Cleared samples were refractive-index-matched using RapiClear (Sunjin Labs) and imaged on a custom-built light-sheet microscope^62^ using a 10× objective and 5-µm step size or an LaVision Ultramicroscope with a 0.63× zoom macro lens with a step size of 5 µm. Images were visualized using Vision4D (Arivis).

For automated whole-brain registration and cell-segmentation analysis, images were loaded onto Arivis Vision4D software, and neurons were segmented using a built-in supervised pixel-based classifier package based on Ilastik^63^ (‘Trainable Segmenter’). Segmentation masks were converted to binary cell masks. Raw light-sheet microscopes images and cell masks were registered to a common reference space defined by the Allen Institute’s Reference Atlas and analysed in a region-based manner using our MIRACL package^40^.

### Induction of *Fos* after pacing

Mice were injected retro-orbitally with AAV9-mTNT-ChRmine-oScarlet or vehicle at three to four weeks of age. At four weeks after injection, mice were handled and acclimatized to fresh clean cages and optical-pacing equipment for a minimum of seven days before pacing experiments. On the day of labelling, mice were allowed to acclimatize to optical-pacing equipment for at least 2 h in a fresh clean cage with food and water, stimulated for 15 min and euthanized 30 min after stimulation by perfusion with ice-cold PBS and 4% PFA under heavy anaesthesia. Tissue was post-fixed in 4% PFA on ice for an additional 24 h (brain) before staining and imaging.

### In situ hybridization

Post-fixed brains were cut with a vibratome into 65-µm coronal slices. Heart and other organs were sliced at 200-µm thickness. Tissue slices were stored in 70% ethanol at −20 °C. Established protocols for third-generation hairpin chain reaction (HCR) in situ hybridization were used for coronal slice^64^. In situ hybridization probes (ChRmine, *Fos* and *Slc6a2*) were designed by and purchased from Molecular Instruments. Hybridization was performed overnight in hybridization buffer (Molecular Instruments) at 4 nM probe concentration. The next day, slices were washed (three times in wash buffer at 37 °C then twice in 2× SSCT at room temperature; 30 min each) and then incubated in amplification buffer. Dye-conjugated hairpins (B1-647, B3-488 and B5-546) were heated to 95 °C for 1 min and then cooled to 4 °C. Hairpin amplification was performed by incubating individual slices in 50 µl of amplification buffer with B1, B3 and B5 probes at concentrations of 240 nM overnight in the dark. Samples were stained with DAPI, washed three times with 5× SSCT for 30 min each and then equilibrated in exPROTOS (125 g iohexol, 3 g diatrizoic acid and 5 g *N*-methyl-d-glucamine dissolved in 100 ml deionized water with the refractive index adjusted to 1.458) (ref. ^65^), a high-refractive-index mounting solution, then imaged. Slices were imaged on a confocal microscope (Olympus FV3000).

### Cardiac histology

At 48 h post-fixation, hearts were sectioned into 200-µm slices. For staining, slices were first incubated for 10 min in blocking solution (3% normal donkey serum (NDS) in PBST), followed by primary antibody staining overnight at 4 °C using the following antibodies: anti-vimentin (ab24525), anti-cardiac troponin I (ab188877) or anti-PGP9.5 (ab108986), purchased from Abcam at 1:200 dilution in blocking solution. Slices were then washed twice in PBST, then stained with secondary antibodies (1 mg ml^−1^) at 1:500 dilution for 3 h at room temperature using the following: F(ab’)2 anti-chicken 488 (703-546-155) and anti-rabbit 647 (711-606-152) purchased from Jackson ImmunoResearch Laboratories. The slices were then stained with DAPI and washed three times with PBST (30 min per wash). Sections were mounted onto slides and mounted with exPROTOS. Slices were imaged on a confocal microscope (Olympus FV3000).

### Stereotaxic surgery for optogenetic experiments

For all surgeries, mice were anesthetized with 1–2% isoflurane, and placed in a stereotaxic apparatus (Kopf Instruments) on a heating pad (Harvard Apparatus). Fur was removed from the scalp, the incision site was cleaned with betadine and a midline incision was made. Sterile surgical techniques were used, and mice were injected with sustained-release buprenorphine for post-operative recovery. Mice were allowed to recover for at least two weeks after surgery before behavioural experiments.

For intracranial optogenetic experiments, virus was injected using a 33-gauge beveled needle and a 10-µl Nano-fil syringe (World Precision Instruments), controlled by an injection pump (Harvard Apparatus). Five hundred nanolitres of AAVdj-hSyn::iC++-eYFP or AAVdj-hSyn::eYFP (5×10^11^ vg ml^−1^) was injected at 150 nl per min and the syringe was left in place for at least 10 min before removal. The following coordinates were used (relative to Bregma): posterior insula (−0.58 (anterior–posterior (AP)), ±4.2 (medial–lateral (ML)), −3.85 (dorsal–ventral (DV)); mPFC (1.8 (AP), ±0.35 (ML), −2.9 (DV)). Optical fibres (0.39 NA, 200 µm; Thorlabs) were implanted 200 µm above virus injection coordinates. Fibres were secured to the cranium using Metabond (Parkell). Mice were allowed to recover for at least two weeks before behavioural testing.

### In vivo electrophysiology

The mice with or without cardiac-targeted ChRmine expression were implanted with custom-made headplates, reference electrodes and cyanoacrylate-adhesive-based ‘clear-skull caps’ as previously described^66^. After recovery, mice were water-restricted and habituated to head fixation, but they were allowed to drink water to satiate thirst before recording sessions. Craniotomies were made with a dental drill at least several hours before recording sessions and were sealed with Kwik-Cast (World Precision Instruments). Exposed craniotomies before, during and after recordings were kept moist with frequent application of saline until sealed with Kwik-Cast.

Before recordings, the mice were placed into the pacemaker vests and reliable pacing was confirmed by ECG under brief anaesthesia with isoflurane. Then the mice were head-fixed and allowed to recover. Next, one or two (for simultaneous bilateral recordings) four-shank Neuropixels 2.0 probes mounted on a multi-probe manipulator system (New Scale Technologies) and controlled by SpikeGLX software (Janelia Research Campus) were inserted through the craniotomies at variable angles (0–20°) depending on the recording geometry. Typically the probes were aimed to touch the skull around the insula, which could be inferred from probe bending or changes in local field potential, and then were retracted around 100 µm and allowed to sit in place for at least 15 min before recordings. Recordings were performed along each of the four shanks sequentially while mice received 5 s of optical stimulation (900 bpm (15 Hz)) with inter-trial intervals of at least 15–25 s. Probes were cleaned with trypsin between recording sessions. Spike sorting was performed by Kilosort 2.5 and auxiliary software as previously described^66^.

After recordings, the brains were perfused, cleared, imaged and registered to the Allen Brain Atlas as previously described^66^. Using the traces of lipophilic dye CM-DiI or DiD (which coated the probes before each insertion) and electrophysiological features, the atlas coordinates of the recorded single units were determined.

The spikes from single units were aligned to pacing onset, and the visualized peri-stimulus time histograms were calculated by subtracting 5 s baseline firing rate, 10 ms binning and 500 ms half-Gaussian filtering. The population-averaged firing rate of each region was calculated by combining *z*-scores (before filtering) over time for all single units in the region of interest. Specifically, we used hierarchical bootstrap to combine data from multiple levels as previously described^66^. For each condition, 100 bootstrap datasets were generated, and their mean and s.d. represented the mean and s.e.m. of the initial dataset. For statistical tests comparing ChRmine and control groups, the one-sided *P* value for the null hypothesis (the ChRmine firing rate subtracted by the control firing rate is zero) was calculated as the fraction of these subtracted values from the pairs of the resampled means (averaged over the time window of interest) that were smaller than zero.

### Optogenetic freely moving behaviour

For optogenetic inhibition of iC++, a 473-nm laser (Omicron Laserage) was used to deliver constant light at 2–3 mW measured from the tip. Laser shutters were controlled using a Master-8 receiving synchronized input from behaviour apparatus and control software (Ethovision).

### Statistical analysis

The target number of subjects used in each experiment was determined on the basis of numbers in previously published studies. No statistical methods were used to predetermine sample size or randomize. Criteria for excluding mice from analysis are listed in the methods. Mean ± s.e.m. was used to report statistics. The statistical test used, definition of *n* and multiple-hypothesis correction where appropriate are described in the figure legends. Unless otherwise stated, all statistical tests were two-sided. Significance was defined as alpha = 0.05. All statistical analyses were performed in GraphPad Prism 9.

### Reporting summary

Further information on research design is available in the Nature Portfolio Reporting Summary linked to this article.

## Online content

Any methods, additional references, Nature Portfolio reporting summaries, source data, extended data, supplementary information, acknowledgements, peer review information; details of author contributions and competing interests; and statements of data and code availability are available at 10.1038/s41586-023-05748-8.

## Supplementary information

Reporting Summary

Supplementary Video 1 **In vitro** **optical pacing of cardiomyocytes**. Representative brightfield movie of contracting cardiomyocytes expressing ChRmine. Optical stimulation (light ON) was applied at 5 Hz, with 10-ms pulse width at 585 nm.

## Data Availability

All primary data for all figures and extended data figures are available from the corresponding author upon request.
