# Supplementary material for: Cardiogenic control of affective behavioural state
Source: Nature. 2023 Mar 1;615(7951):292–9. doi: 10.1038/s41586-023-05748-8 (PMC9995271; doi:10.1038/s41586-023-05748-8)
Supplement: Supplementary file 1 — Reporting Summary [file 41586_2023_5748_MOESM1_ESM.pdf]

nature portfolio

Corresponding author(s): Karl Deisseroth

Last updated by author(s): Dec 19, 2022

Reporting Summary

Nature Portfolio wishes to improve the reproducibility of the work that we publish. This form provides structure for consistency and transparency in reporting. For further information on Nature Portfolio policies, see our Editorial Policies and the Editorial Policy Checklist.

Statistics

For all statistical analyses, confirm that the following items are present in the figure legend, table legend, main text, or Methods section.

n/a	Confirmed	
☐	☒	The exact sample size (n) for each experimental group/condition, given as a discrete number and unit of measurement
☐	☒	A statement on whether measurements were taken from distinct samples or whether the same sample was measured repeatedly
☐	☒	The statistical test(s) used AND whether they are one- or two-sided<br>*Only common tests should be described solely by name; describe more complex techniques in the Methods section.*
☐	☒	A description of all covariates tested
☐	☒	A description of any assumptions or corrections, such as tests of normality and adjustment for multiple comparisons
☐	☒	A full description of the statistical parameters including central tendency (e.g. means) or other basic estimates (e.g. regression coefficient) AND variation (e.g. standard deviation) or associated estimates of uncertainty (e.g. confidence intervals)
☐	☒	For null hypothesis testing, the test statistic (e.g. F, t, r) with confidence intervals, effect sizes, degrees of freedom and P value noted<br>*Give P values as exact values whenever suitable.*
☒	☐	For Bayesian analysis, information on the choice of priors and Markov chain Monte Carlo settings
☒	☐	For hierarchical and complex designs, identification of the appropriate level for tests and full reporting of outcomes
☒	☐	Estimates of effect sizes (e.g. Cohen's d, Pearson's r), indicating how they were calculated

Our web collection on statistics for biologists contains articles on many of the points above.

Software and code

Policy information about availability of computer code

Data collection	Olympus Fluoview FV3000 (confocal image acquisition), Ethovision XT14 (behavioral tracking), Open Ephys (extracellular in vivo recording acquisition), MATLAB R2021b, SpikeGLX (neuropixels electrophysiology), Rodent Surgical Monitor+ Indus Instruments (Electrocardiography)
Data analysis	MATLAB R2021b, Python, ImageJ 1.53, Origin 2020, GraphPad Prism, Kilosort2.5, Phy2, Arivis Vision4D, MIRACL package for whole-brain registration, Ilastik for cell segmentation, Vision4D (Arivis)

For manuscripts utilizing custom algorithms or software that are central to the research but not yet described in published literature, software must be made available to editors and reviewers. We strongly encourage code deposition in a community repository (e.g. GitHub). See the Nature Portfolio guidelines for submitting code & software for further information.

Data

Policy information about availability of data

All manuscripts must include a data availability statement. This statement should provide the following information, where applicable:

- Accession codes, unique identifiers, or web links for publicly available datasets
- A description of any restrictions on data availability
- For clinical datasets or third party data, please ensure that the statement adheres to our policy

All primary data for all figures and supplementary figures are available from the corresponding author upon request.

Human research participants

Policy information about studies involving human research participants and Sex and Gender in Research.

Reporting on sex and gender	N/A
Population characteristics	N/A
Recruitment	N/A
Ethics oversight	N/A

Note that full information on the approval of the study protocol must also be provided in the manuscript.

Field-specific reporting

Please select the one below that is the best fit for your research. If you are not sure, read the appropriate sections before making your selection.

☒ Life sciences ☐ Behavioural & social sciences ☐ Ecological, evolutionary & environmental sciences

For a reference copy of the document with all sections, see nature.com/documents/nr-reporting-summary-flat.pdf

Life sciences study design

All studies must disclose on these points even when the disclosure is negative.

Sample size	No statistical methods were used to determine sample size a priori. We chose sample sizes there were similar to those reported in our previous publications.
Data exclusions	Criterias for animal exclusion were pre-established. Animals with unintended experimental error were excluded based on poor viral expression from mis-injection or misplacement of fiber optics. Otherwise, no data were excluded.
Replication	Data was collected in multiple experiments (at least twice) and all results were pooled. No experiments failed to replicate. Behavior that required repeated measure were counter balanced.
Randomization	Mice were randomly assigned to each experimental group.
Blinding	Experimenters were not blinded to opsin identity during behavioral experiments because animals were run across several behavioral tasks, some of which required long training periods. Furthermore, electrophysiology recordings were performed in a difficult-to-target region, so opsin identity needed to be known in order to guarantee the best recording outcome. Experimenters were blinded to treatment conditions for in situ Fos and other histology quantification.

Reporting for specific materials, systems and methods

We require information from authors about some types of materials, experimental systems and methods used in many studies. Here, indicate whether each material, system or method listed is relevant to your study. If you are not sure if a list item applies to your research, read the appropriate section before selecting a response.

Materials & experimental systems

n/a	Involved in the study	
☐	☒	Antibodies
☒	☐	Eukaryotic cell lines
☒	☐	Palaeontology and archaeology
☐	☒	Animals and other organisms
☒	☐	Clinical data
☒	☐	Dual use research of concern

Methods

n/a	Involved in the study	
☒	☐	ChIP-seq
☒	☐	Flow cytometry
☒	☐	MRI-based neuroimaging

Antibodies

Antibodies used	Abcam: Anti-Vimentin (ab24525, 1:200), Anti-Cardiac Troponin I (ab188877, 1:200), or Anti-PGP9.5 (ab108986, 1:200). Jackson ImmunoResearch: F(ab')2 Anti-Chicken 488 (703-546-155, 1:500) and Anti-Rabbit 647 (711-606-152, 1:500).

Validation	Antibody validation was performed by comparing immunofluorescence signal with established literature. Antibodies have also been previously validated (e.g. vendor, other labs).

Animals and other research organisms

Policy information about studies involving animals; ARRIVE guidelines recommended for reporting animal research, and Sex and Gender in Research

Laboratory animals	C57BL/6J mice (Jackson Laboratories, Strain #000664, 4-12 week male or female). Fos2A-iCreER (TRAP2; JAX #030323) and B6;129S6-Gt(ROSA)26Sortm14(CAG-tdTomato)/Hze/J (Ai14; JAX #007908) were crossed to generate double transgenicTRAP2 mice (4-12 week male or female mice were used for this study).
Wild animals	No wild animals were used in this study.
Reporting on sex	Analysis on both sexes were reported in this study.
Field-collected samples	No field samples were used in this study.
Ethics oversight	All experimental protocols were approved by the Stanford University Institutional Animal Care and Use Committee (IACUC) following the National Institutes of Health guidelines for the care and use of laboratory animals.

Note that full information on the approval of the study protocol must also be provided in the manuscript.
